# Contributions to the Ohio College of Dental Surgery

**Published:** 1849-07

**Authors:** 


### CONTRIBUTIONS TO THE OHIO COLLEGE OF DENTAL SURGERY.

We acknowledge the receipt of a specimen of artificial work, by Dr. Brown, our colleague, of St. Louis. What makes it the more valuable is, that it was made for a patient, who not having called for the teeth, they were sent to enrich the museum of the Dental College. The specimen consists of four Incisors above, mounted on good gold plate; and the entire job finished off in a very neat style, shewing evidently that the Doctor has not neglected the mechanical part of our profession. We would be glad to receive not only such specimens of skill, but Dental and Medical Books, and anything that might be of interest to the dental student or profession. All such are filed away, and carefully preserved with the donors' names.—[CIN. ED.

